# Caspofungin for treatment of invasive aspergillosis in Germany: results of a pre-planned subanalysis of an international registry

**DOI:** 10.1186/2047-783X-17-7

**Published:** 2012-04-17

**Authors:** Gerlinde Egerer, Dietmar Reichert, Mathias W Pletz, Peter Kaskel, Karl J Krobot, Johan Maertens

**Affiliations:** 1 Ruprecht-Karls-Universität Heidelberg, Universitaetsklinikum, Medizinische Klinik Abteilung V, Heidelberg, Germany; 2 Klinikum Oldenburg gGmbH, Oldenburg, Germany; 3 Medizinische Hochschule Hannover, Hanover, Germany; 4 Outcomes Research, MSD SHARP & DOHME GMBH, Haar, Germany; 5 4UZ Gasthuisberg, Leuven, Belgium; 6 Hämatologisch-Onkologische Gemeinschaftspraxis, Westerstede, Germany; 7 Division of Gastroenterology, Hepatology and Infectious Diseases, Jena University Hospital, Jena, Germany

**Keywords:** Invasive fungal disease, Invasive aspergillosis, Therapy, Echinocandins, Caspofungin

## Abstract

**Background:**

This study is a pre-planned country-specific subanalysis of results in Germany from a multinational multicenter registry to prospectively assess real-world experience with caspofungin administered for treatment of proven or probable invasive aspergillosis (IA).

**Methods:**

Data from patients treated with caspofungin for a single episode of IA were collected. Effectiveness was determined by the local investigator as favorable (complete or partial response) or unfavorable (stable disease, failure or death) at the end of caspofungin therapy. Descriptive statistics with binomial exact confidence intervals were employed.

**Results:**

Forty-two consecutive patients were identified in three German centers. Three patients (7%) had proven IA and 39/42 (93%) had probable IA (modified European Organization for Research and Treatment of Cancer/Mycosis Study Group (EORTC/MSG) criteria). Forty-one patients had pulmonary IA and one had tracheal IA. Caspofungin monotherapy was received by 36/42 patients (86%); of these, 26/36 (72%) received salvage therapy. A favorable response was observed in 29/42 patients (69%; 95% CI 53 to 82%); of these, 21/29 (72%) had a complete and 8/29 (28%) a partial response. Favorable response rate was 69% in patients with monotherapy (95% CI 52% to 84%; 25/36 patients), and 67% in patients receiving combination therapy (95% CI 22% to 96%; 4/6 patients). Favorable response rate in patients with first line therapy was 64% (95% CI 31% to 89%; 7/11 patients), and 73% in patients with second line therapy (95% CI 54% to 88%; 20/30 patients). No adverse events were reported. In total, 35/42 patients (83%; 95% CI 69 to 93%) survived seven days after completion of caspofungin therapy.

**Conclusions:**

These real-life findings in Germany are consistent with the international findings from this registry and with findings from randomized studies.

## Introduction

Invasive aspergillosis poses a major threat to patients with hematologic diseases and can greatly jeopardize the success of treatment of the underlying condition [1]. Among patients with hematologic and oncologic conditions, patients who have undergone stem cell transplantation form a particularly high risk group for invasive aspergillosis. Other risk factors are major surgical procedures, HIV/AIDS, cancer, immunosuppressive therapy and advanced age [2]. Invasive aspergillosis is associated with mortality rates of 30% to 90% [3], and the one-year survival rate of patients with fungal infections following stem cell transplantation has been reported to be as low as 20% in some cases. Donhuijsen *et al*. found invasive mycosis to be present in 340 of 1,591 autopsied patients with hematologic neoplasias, the proportion of aspergillosis among these cases rising from 28% in the period 1976 to 1980 to 89% in the period 2001 to 2005 [4].

Current guidelines recommend treatment with azoles, polyenes and echinocandins [1,5]. Caspofungin is the only echinocandin presently licensed in Europe for the treatment of invasive aspergillosis, being approved for the treatment of invasive aspergillosis in adult and pediatric patients who are refractory to or intolerant of, for example, amphotericin B and as empirical therapy for presumed fungal infections (such as *Candida *or *Aspergillus) *in febrile neutropenic adult or pediatric patients [6].

The purpose of the registry was to collect data on the use of caspofungin in everyday clinical practice in patients with invasive aspergillosis. As data from Germany on this disease have not previously been available, we present here the results of a pre-planned national subanalysis.

## Methods

Patients treated in hospitals in Australia, Belgium, Brazil, Germany, Greece, Jordan, Korea, Russia, Singapore, Slovenia and Taiwan were consecutively included in the international registry in the period from April 2006 to September 2007 [7].

The study was submitted to the responsible ethics committees in accordance with local regulations. Inclusion criteria were informed consent by the patient, age at least 16 years, a diagnosis of proven or probable invasive aspergillosis, mono- or combination therapy with caspofungin, and non-participation in any clinical trial on antimycotic therapy sponsored by MSD or Merck & Co. The infection was classified as invasive aspergillosis by the treating physician on the basis of the individual clinical assessment and local diagnostic and treatment standards. As an aid to classification of the *Aspergillus *infection as probable or proven, the treating physicians were provided with the criteria of the European Organisation for Research and Treatment of Cancer/Mycoses Study Group of the year 2002 [8]. There were no exclusion criteria.

No study medication was supplied in connection with this registry. Dosage in the individual patient was determined by the physician on the basis of the drug prescribing information [6] and local treatment standards. One treatment cycle with caspofungin was documented for each patient. Patients included in the registry were observed from the start of their period of hospitalization until their discharge or death. The decision to treat was taken by the treating physician alone on the basis of the patient's clinical situation.

The data collected consisted of patient data (for example, gender, weight and age), previous illnesses, risk factors and information on caspofungin therapy, such as date commenced, duration, dose, response and previous treatment. Also collected were data on the patient's general condition, neutropenic status, liver function and organ involvement; microbiologic findings; drug interactions; safety; and concomitant antimycotic medication. At the end of treatment data on survival, clinical condition, results of imaging investigations, microbial resistance and efficacy were collected.

The objective was to assess real-world effectiveness on the basis of an assessment of individual response to treatment undertaken by the treating physician at the end of caspofungin administration in accordance with standardized criteria [9], whereby only complete or partial remission was deemed to constitute a response. A complete response to treatment with caspofungin was deemed to be present if in the opinion of the treating physician all clinical signs and symptoms of IA and all radiologic and bronchoscopic abnormalities had resolved completely by the end of treatment. A partial response required a clinically significant improvement in all clinical signs and symptoms consistent with IA and a pronounced improvement in radiologic (at least 50%) and bronchoscopic abnormalities, and was considered to be present - independent of the extent of clinical or radiologic improvement - even in the presence of persistent radiologic changes. Non-response to treatment was defined as stable disease, treatment failure or death.

For the assessment of safety, the physicians were required to report serious and non-serious clinical and laboratory adverse events that were possibly, probably or definitely related to the caspofungin therapy. Serious adverse events were defined as adverse events that were life-threatening; that resulted in death or hospitalization or prolongation of a period of hospitalization; or that led to inability to work; and included congenital anomalies, cancers and all complications, which, from a medical point of view, are to be regarded as serious. An independent expert panel reviewed the analysis of the entire international data for accuracy and completeness [7].

The analysis was descriptive. Binomial exact 95% confidence intervals (CIs) were calculated with the aid of SAS PROC FREQ.

## Results

Data were collected from 42 patients from three centers in Germany (out of a worldwide total of 103 patients); 43% of the patients (18/42) were female and 57% (24/42) male. Median patient age at onset of disease was 57 years (range 17 to 75 years). Median weight was 74 kg (range 44 to 98 kg). Underlying dísease was active malignancy in 39/42 patients (93%; Acute myeloic leukemia - 28, lymphoma - 4, myeloma - 3, acute lymphatic leukemia - 2, breast cancer - 1, other - 1), and organ transplantation in 3/42 patients (7%). The patients suffered from a median of four comorbidities, for example, 16/42 (38%) had pulmonary disease; and 10/42 (24%) had arterial hypertension, or heart disease, respectively. History-taking revealed a median of five risk factors, for example, before receiving caspofungin 41/42 patients (98%) had received broad-spectrum antibiotics and 39/42 patients (93%) immunosuppressants. For further information, see Table 1.

**Table 1:** Demographic data and patient characteristics

Variable	Caspofungin monotherapy (N = 36)	Caspofungin combination therapy (N = 6)	Total (N = 42)
Gender, n (%)			
Male	22 (61%)	2 (33%)	24 (57%)
Female	14 (39%)	4 (67%)	18 (43%)
Age (years; median (range))	59 (20 to 75)	42 (17 to 57)	57 (17 to 75)
Affected organ, n			
Lung	35	6	41
Trachea	1	0	1
Neutropenic status at start of caspofungin therapy			
Neutrophilic granulocytes < 500/μL	27 (75%)	1 (17%)	28 (67%)
Neutrophilic granulocytes ≥ 500/μL	9 (25%)	5 (83%)	14 (33%)
No. of risk factors per patient (median (range))	5 (3 to 9)	7 (3 to 7)	5 (3 to 9)
Risk factors, n (%)*'			
Active cancer	35 (97%)	4 (67%)	39 (93%)
Immunosuppressive therapy	34 (94%)	5 (83%)	39 (93%)
Neutropenia at hospitalization	26 (72%)	1 (17%)	27 (64%)
Allogeneic HSCT	5 (14%)	3 (50%)	8 (19%)
Prior colonization with fungi	5 (14%)	1 (17%)	6 (14%)
Acute renal disease	4 (11%)	1 (17%)	5 (12%)
Diabetes mellitus	4 (11%)	1 (17%)	5 (12%)
Autologous HSCT	4 (11%)	0	4 (10%)
AIDS/HIV disease	3 (8%)	0	3 (7%)

*Odered by frequency. 'Multiple answers possible. *HSCT *hematopoetic stem cell transplantation

The standard caspofungin regimen, that is, 70 mg on Day 1 and 50 mg from Day 2, was given in 38/42 patients (90%). One patient received a lower dosage (50 mg on Day 1 and 30 mg from Day 2) and three patients a higher dosage (70 mg on Day 1 and 70 mg from Day 2). Information on the use of caspofungin mono- and combination therapy, and pretreatment with antimycotic agents, is given in Table 2. The median duration of caspofungin treatment was 11 days (mean 18.4, range 5 to 152, n = 42) for all patients and 11 days (mean 13.7, range 5 to 38; n = 36) for the monotherapy group.

**Table 2:** Patients treated with caspofungin

Type of use	Monotherapy (N = 36)	Combination therapy (N = 6)	Total (N = 42)
Caspofungin first-line therapy*	10 (28%)	1 (17%)	11 (26%)

Caspofungin second-line therapy**	26 (72%)	5 (83%)	31 (74%)
*Thereof reason for switching to caspofungin*			
Clinically refractory to first-line therapy	21 (58%)	5 (83%)	26 (62%)
Toxicity with first-line therapy	2 (6%)	0	2 (5%)
Other**	3 (8%)	0	3 (7%)

*The reason for using caspofungin as the first line therapy was "probable aspergillosis" in 10 patients and "proven aspergillosis" in 1 patient.

**In 25 patients, this consisted of azoles (9 posaconazole, 7 voriconazole, 6 fluconazole (of these, 5 for prophylactic use and 1 for unknown reasons), 3 itraconazole), in 5 patients polyenes (2 amphotericin colloidal dispersion, 3 liposomal amphotericin B), and in 1 patient other antimycotics. Prior antimycotic therapy had been given for a mean duration of 13.2 days.

**Breakthrough infection during azole prophylaxis

The treating physicians considered that all the patients were suffering from invasive aspergillosis, this diagnosis being regarded as proven in 3/42 patients (7%; in 2 patients *Aspergillus fumigatus*, in 1 patient not further specified) and probable in 39/42 patients (93%; diagnosis in these case was based on the investigator's judgment). Pulmonary involvement was present in 41/42 patients (98%) and tracheal involvement in 1patient. The absolute neutrophil count at the start of treatment was < 500 in 28/42 patients (67%). Prior antimycotic therapy had been given in 31/42 patients (74%) for a mean duration of 13.2 (SD 7.9) days. For further information, see Table 2.

The treating physicians considered that 29/42 patients (69%; 95% CI 53 to 82%) responded to treatment. Of these, 21/29 patients (72%) showed a complete response and 8/29 patients (28%) a partial response. In 10 patients, the disease remained stable. One patient, who had received prior treatment with liposomal amphotericin B, was classified by the treating physician as showing treatment failure after eight days of caspofungin therapy. A patient with AML, who had received prior prophylactic therapy with fluconazole for four days followed by six days of treatment with caspofungin and then three days of treatment with voriconazole, died of cerebral aspergillosis. Data on real-world effectiveness are absent for one patient who received combination therapy. Further details are given in Table 3. The survival rate seven days after the end of caspofungin therapy was 83% overall (35/42 patients; 95% CI 69 to 93%) and 89% (25/28 patients; 95% CI 75 to 97%) in patients with neutropenia at the start of caspofungin therapy. A total of 34/42 patients (81%) were alive at hospital discharge, of whom 28 patients were discharged home, and 6 were discharged to another institution. A total of 7/42 patients (17%) had died in hospital, and there was one patient with unknown status at hospital discharge (2%).

**Table 3:** Real-world effectiveness of treatment as assessed by the treating physician (n = 41/42*)

Variable	Response, % (n/N) [95% CI]
Overall	70.7 (29/41) [54.5 to 83.9]

Probable aspergillosis	71.8 (28/39) [55.1 to 85.0]
Proven aspergillosis	50.0 (1/2) [37.4 to 74.5]

Combination therapy**	80.0 (4/5) [28.4 to 99.5]
Monotherapy	69.4 (25/36) [51.9 to 83.7]

First-line therapy***	63.6 (7/11) [30.8 to 89.1]
Second-line therapy	73.3 (20/30) [54.1 to 87.7]

Neutropenic status at start of caspofungin therapy	
Neutrophilic granulocytes < 500/uL	71.4 (20/28) [51.3 to 86.8]
Neutrophilic granulocytes ≥ 500/uL	69.2 (9/13) [38.6 to 90.9]

Risk factors****	
Allogeneic HSCT	87.5 (7/8) [47.3 to 99.7]
Acute renal disease	80.0 (4/5) [28.4 to 99.5]
Neutropenia at hospitalization	70.4 (19/27) [49.8 to 86.2]
Active cancer	69.2 (27/39) [52.4 to 83.0]
Immunosuppressive therapy	68.4 (26/38) [51.3 to 82.5]
Prior colonization with fungi	66.7 (4/6) [22.3 to 95.7]
Autologous HSCT	50.0 (2/4) [6.8 to 93.2]

* No data were available for one lung transplant patient with proven aspergillosis (*A. fumigatus*), who had received triple combination therapy (amphotericin B, caspofungin, voriconazole). If this patient is regarded as a failure, the response rate for all 42 patients is: favorable response, 29/42 patients (69%; 95% CI 53 to 82%).

** Combination of caspofungin with amphotericin B desoxycholate (two patients), with fluconazole (one patient), with posaconazole (one patient), with amphotericin B desoxycholate plus voriconazole (one patient). Note, if the patient described in * is included in the effectiveness population and (*post hoc*) counted as a failure, the response rate for patients with combination therapy is 66.7% (4/6) and the 95% confidence interval is 22.2% to 95.7%.

*** All patients receiving caspofungin first line were on monotherapy.

**** Multiple answers possible.

In no patient was the caspofungin therapy stopped because of a drug interaction between caspofungin and another antifungal agent. The treating physicians considered that there were no serious or non-serious clinical or laboratory adverse events possibly, probably or definitely related to the caspofungin therapy. A total of 28 patients were discharged home after the end of their period of hospitalization and six patients were transferred to another institution. Seven patients died during the observation period; in none of these cases did the treating physician consider there to be a causal relationship with the antifungal therapy.

## Discussion

In a randomized double-blind study by Walsh *et al*. [10] on the use of caspofungin vs. liposomal amphotericin B as empirical therapy in neutropenic patients with suspected invasive fungal disease, 24 cases of pre-existing invasive aspergillosis that were unrecognized at the start of treatment were identified, with 12 such cases in each treatment arm. The response rate was 42% in the caspofungin arm and 8% in the liposomal amphotericin B arm. The response rates for caspofungin reported in non-randomized studies are in the range 33 to 56% [9,11-13]. In our group of patients the use of caspofungin under real-world conditions in Germany likewise led to high response and survival rates; whereas, the response rate in patients with proven or probable IA in the international study, of which this forms a part, was 56% (57/101; 95% CI 47 to 66%), a figure that is within the range of previously published results [7]. The response rate in the subanalysis for Germany was higher, namely 69% (29/41; 95% CI 54 to 84%). Technical, social and psychological standards and conditions in health care may vary across countries and sites and impact on diagnostic accuracy. On the other hand, patients in Germany were treated much earlier than the patients in other countries, as evidenced by the fact that the proportion of patients with proven aspergillosis was only 7% in the German population compared to 30% internationally. The Infectious Diseases Working Party (AGIHO) of the German Society for Hematology and Oncology (DGHO) recently issued a BIII recommendation advising early initiation of treatment at the first sign of IA, as this improves the probability of survival [1]. The licensed first-line treatments for invasive aspergillosis likewise show higher response rates when used earlier. For example, in an unblinded study on primary therapy of invasive aspergillosis with voriconazole, 30/67 patients (45%) with definite invasive aspergillosis responded to treatment compared to 46/66 patients (60%) with probable aspergillosis (defined as the presence of a halo or cavitation with an air-crescent sign in a patient with neutropenia or allogeneic SCT) [14]. In a double-blind study on primary therapy of invasive aspergillosis, Cornely *et al*. investigated the use of liposomal amphotericin B at different dose levels (3 mg vs. 10 mg/kg bodyweight) [15]. The overall response rate in the 107 patients who received the licensed dose of 3 mg/kg bodyweight was 50%. Interestingly, the diagnosis was considered to be probable (defined as the presence of a halo or cavitation with an air-crescent sign in a patient with neutropenia or allogeneic SCT) in 99/107 patients and proven in only 8/107 patients. In our study, 11/42 patients in Germany (26%) received caspofungin as primary therapy (in one case as part of combination therapy) compared to 20% of patients internationally. These patients who received primary therapy with caspofungin showed a response rate of 64% compared to 60% internationally. Unlike the patients from other countries, none of the patients from Germany had *Aspergillus *fungemia. Most (74%) of our patients were given caspofungin as salvage therapy. Caspofungin is licensed for use as second-line therapy of invasive aspergillosis and is given an AII recommendation for this purpose in the current AGIHO guidelines. In addition, the guidelines of the European Conference on Infections in Leukemia (ECIL) give caspofungin a CII recommendation for primary therapy of invasive aspergillosis, whereas it gives voriconazole a recommendation of AI or CIII (when initiated orally) and liposomal forms of amphotericin B a BI or BII recommendation, respectively [5]. One possible reason why caspofungin was used in the patients considered here may be the high potential that azoles have for interaction with modern oncologic therapies - in particular with vinca-alkaloids - because of cytochrome P450 metabolism [1]. Also notable in this regard is the hepatotoxicity and nephrotoxicity of the polyenes [16,17]. Interestingly, in the patients in our series caspofungin was used primarily because of non-response to, and/or toxicity of, previous treatment. The treating physicians did not report any adverse events possibly, probably or definitely related to caspofungin therapy.

The patients in this series were severely ill patients in whom it was, therefore, especially important to ensure successful treatment of the underlying condition: 79% were suffering from a malignant or hematologic disease, 93% were receiving immunosuppressive therapy, 98% had recently received a broad-spectrum antibiotic, 64% were neutropenic and 45% had a central venous catheter in place. This circumstance may also explain why a proportion of patients (14%) were given combination therapy despite the paucity of evidence to support such treatment. Whereas the average duration of caspofungin treatment in this real-life setting was at the lower end of what has been reported in earlier studies, the total range reported here is completely in line with what previously has been reported [9,11-13].

Synergistic effects among echinocandins and amphotericin B and azoles have been demonstrated *in vitro *[18]. Recently Caillot *et al*. [19] reported the first evidence from a randomized study that use of liposomal amphotericin B (3 mg/kg) in combination with caspofungin in invasive aspergillosis achieves a higher response rate at the end of treatment than does high-dose liposomal amphotericin B alone, whereby the combination showed good tolerance and achieved a 12-week survival of 100%. Further studies are required to confirm the value of combination therapy in invasive aspergillosis.

The limitations of the present study include its one-armed observational design. Also, the assessment of real-world effectiveness was based exclusively on evaluation by the treating physician and local diagnostic and treatment standards, and information regarding the rationale for the use of the pre-caspofungin first line treatment is limited. The classification of invasive aspergillosis was based on the 2002 EORTC/MSG criteria [8], and nearly all the *Aspergillus *infections (93%) had been classified by the treating physician as probable and were not reviewed by a panel. Moreover, patients who show signs that are highly suggestive of *Aspergillus *infection could also be classified as having probable invasive aspergillosis. The small number of patients resulted in generally broad confidence intervals. No deaths associated with the caspofungin therapy were reported; however, the causes of the deaths that occurred were not determined. The follow-up period after the end of caspofungin therapy was relatively short.

## Conclusions

Based on assessment by the treating physicians, patients in Germany with proven or probable invasive aspergillosis showed an overall response rate of 69% to caspofungin therapy. The side-effect and interaction profiles were favorable. These results were obtained in a patient population composed largely of high-risk patients with active cancer or neutropenia. The results of this pre-planned Germany-specific analysis are consistent with the results obtained in the overall cohort and with the results of randomized clinical studies on the use of caspofungin in patients with invasive aspergillosis.

## Abbreviations

AGIHO: Infectious Disease Working Party; CIs: confidence intervals; DGHO: German Society for Hematology and Oncology; ECIL: European Conference on Infections in Leukemia; EORTC/MSG: European Organization for Research and Treatment of Cancer/Mycosis Study Group; HSCT: Hematological Stem Cell Transplantat; IA: Invasive aspergillosis; SCT: Stem Cell Transplantation.

## Competing interests

GE has received research support from MSD Sharp & Dohme GmbH and Schering-Plough, acts as a consultant to MSD Sharp & Dohme GmbH and Essex Pharma, and has received a travel allowance from MSD Sharp & Dohme GmbH and Essex Pharma/Schering-Plough. DR has no potential conflicts of interest to disclose. MWP was, in 2007, a research fellow of MSD SHARP & DOHME GMBH, received lecture fees from Astra-Zeneca, Bayer, Brahms, Gielad, MSD SHARP & DOHME GMBH, Novartis, and Pfizer/Wyeth, and acts as a consultant to Pfizer/Wyeth und Sandoz. PK and KJK are employees of MSD Sharp & Dohme GmbH, the German subsidiary of Merck & Co., Inc., the manufacturer of caspofungin. JM has received research support from Merck/MSD and Pfizer; acts as a consultant to Astellas, Bio-Rad, Merck/MSD, Nektar, Pfizer, Schering-Plough, F2G, and Zeneus/Cephalon; and gives talks for Astellas, Bio-Rad, Merck/MSD, Pfizer, Schering-Plough and Zeneus/Cephalon.

## Authors' contributions

GE and JM have made substantial contributions to conception and design of the study. GE, DR, and MWP have made substantial contributions to acquisition of data. GE, PK, KJK and JM have made substantial contributions to analysis and interpretation of data. GE, MWP, PK, KJK and JM have been involved in drafting the manuscript and in revising the manuscript critically for important intellectual content. All authors read and approved the final manuscript.
